# 128 Mental Health Outcomes in Burn Patients: A Single-site Experience

**DOI:** 10.1093/jbcr/irac012.130

**Published:** 2022-03-23

**Authors:** Sanja Sljivic, Lori Chrisco, Rabia Nizamani, Booker King, Felicia Williams

**Affiliations:** University of North Carolina - Chapel Hill, Chapel Hill, North Carolina; North Carolina Jaycee Burn Center, Chapel Hill, North Carolina; UNC Jaycee Burn Center, Chapel Hill, North Carolina; UNC Jaycee Burn Center, Chapel Hill, North Carolina; UNC Jaycee Burn Center, Chapel Hill, North Carolina

## Abstract

**Introduction:**

Burn injuries are a leading cause of morbidity and mortality among patients worldwide. Many survivors continue to suffer from psychiatric sequalae long after their physical injuries have healed. This may even be more pronounced in groups who have a history of mental health disorders prior to admission. Common pre-injury mental health problems may include substance abuse disorders, as well as affective, psychotic, and personality disorders. The aim of this study was to explore the outcomes of patients with previously diagnosed mental health disorders who were admitted to our Burn Center.

**Methods:**

This was a single-site, retrospective review using our institutional Burn Center registry. All adult patients (18 years or older) admitted to our Burn Center between January 1, 2014 and June 30, 2021 who had a previous history of mental health disorders were included in this study. All adult patients who did not have any previous history of mental health disorders were also included for comparative purposes. Variables of interest included demographics, burn mechanism, length of stay (LOS), cost of hospitalization, and mortality. A p-value of < 0.05 was considered statistically significant for all analyses.

**Results:**

There were 7,976 patients included in this study, with 32% of these patients having a previous diagnosis of mental health disorders. The mean age was 43.5 years, and the mean total body surface area (TBSA) involvement was 5.9%. Both the groups with a history of mental health disorders and those without were predominantly male (63.8% versus 68.0%). Most patients were flame-injured in both groups (44.5% versus 40.9%). The mean LOS for those with mental health disorder history was 14.5 days compared to 8.3 days for those without (p < 0.00001). The overall cost of hospitalization was $133,967 for those with mental health disorder history and $65,993 for those without (p < 0.00001). The overall hospital mortality for those with mental health disorder history was 2.3% and 3.4% for those without (p = 0.007).

**Conclusions:**

Although there was no increase in mortality among patients with pre-existing mental health disorders, we did find that there was an increase in the hospital length of stay, as well as the overall cost of hospitalization. These findings do indicate that individuals with pre-existing mental health disorders do not necessarily have worse outcomes in terms of mortality; however, they may need access to care for longer periods of time, which may contribute to increased medical costs.

